# Associations between lymphovascular space invasion, nodal recurrence, and survival in patients with surgical stage I endometrioid endometrial adenocarcinoma

**DOI:** 10.1186/s12957-019-1620-x

**Published:** 2019-05-10

**Authors:** Ashley E. Veade, Jonathan Foote, Jessie Ehrisman, Gloria Broadwater, Brittany A. Davidson, Paula S. Lee, Angeles Alvarez Secord, Andrew Berchuck, Laura J. Havrilesky

**Affiliations:** 1 Department of Obstetrics and Gynecology, Duke University Medical Center, Box 3084, 200 Trent Drive, Baker House 236, Durham, NC 27710 USA; 2 Division of Gynecologic Oncology, Department of Obstetrics and Gynecology, Duke University Medical Center, Durham, NC USA; 3 Department of Biostatistics and Bioinformatics, Duke University Medical Center, Durham, NC USA

## Abstract

**Objective:**

To investigate the predictive value of lymphovascular space invasion (LVSI) for nodal recurrence and overall survival (OS) in patients with stage I endometrioid endometrial cancer (EC) following surgical staging that included adequate lymph node sampling.

**Methods:**

Retrospective analyses of patients undergoing surgical staging for FIGO stage I endometrioid EC between 1998 and 2015 were performed using an institutional database and the National Cancer Database (NCDB). Using the institutional database, logistic regression modeling identified predictors of nodal recurrence; Cox proportional hazards modeling was used to predict progression-free survival (PFS). Utilizing NCDB, Cox proportional hazards modeling was used to predict OS. The Kaplan-Meier method was used to estimate hazard ratios (HR). Survival curves were compared using the log-rank test.

**Results:**

Among 275 institutional cases, LVSI was present in 48 (17.5%). There were 11 nodal recurrences: 18.8% (9/48) of cases with LVSI had a nodal recurrence compared to 0.88% (2/227) of those without LVSI. In multivariate analysis of institutional data, LVSI was the only significant predictor of nodal recurrence (*p* = 0.002). Among 28,076 NCDB cases, LVSI was present in 3766 (13.5%). In multivariate analysis of NCDB, grade 3, LVSI, and depth of invasion (all *p* <  0.001) were prognostic for OS after adjusting for adjuvant radiation.

**Conclusion:**

LVSI is an independent prognostic factor for nodal recurrence in stage I endometrial cancer with lymph node assessment. LVSI is associated with lower OS in NCDB. Given these findings, adjuvant therapy could be considered in these patients.

## Introduction

Endometrial cancer (EC) is the most common cancer of the female reproductive tract in the USA [1–5]. According to the American Cancer Society, over 60,000 new cases of endometrial cancer were diagnosed and about 10,000 women died from the disease in 2016. The majority of patients present with FIGO stage I endometrioid cancers and do not have lymph node metastases at surgical staging. Despite uterine-limited disease on final pathology, 10–20% of these cancers recur [1, 2, 6]. Relapse usually occurs within 24 months of diagnosis [1, 2, 6]. Advanced age, deep myometrial invasion, higher grade, and lymphovascular space invasion (LVSI) all have consistently been associated with an increased risk of recurrence of early-stage endometrial cancers [1, 2, 4, 5, 7, 8].

LVSI is defined as the presence of cancer in lymphatic and/or vascular spaces within the uterine myometrium [9, 10]. LVSI is an established independent risk factor for pelvic lymph node metastasis and is present in about 15% of early-stage endometrial cancers [1, 2, 5, 7, 9–13]. Several studies, including Gynecology Oncology Group (GOG) 99 and the Post-Operative Radiation Therapy in Endometrial Carcinoma (PORTEC) 1 and 2 randomized trials, found that patients with early-stage EC and LVSI treated with external beam radiation therapy (EBRT) had a decreased risk of pelvic recurrence without significant improvement in OS [6, 8, 10, 14]. Based on these findings, Bosse et al. recommended consideration of adjuvant EBRT in early-stage EC patients with substantial LVSI, especially in the presence of additional risk factors [10]. The GOG 99 and PORTEC trials did not specifically address the relationship between LVSI and recurrence in lymph node basins.

The primary objective of this study was to investigate the relationship between LVSI and nodal basin recurrence among women who underwent surgical staging that included nodal evaluation for stage I endometrioid EC. The secondary objectives were to identify recurrence patterns within these staged LVSI-positive patients and to investigate the relationship between LVSI and PFS and OS.

## Materials and methods

Two separate retrospective cohorts were studied to investigate the relationship between LVSI and cancer outcomes: the Duke University EC database and the National Cancer Database (NCDB). Two cohorts were utilized in order to evaluate both overall survival and nodal recurrence.

### Institutional retrospective cohort study

The Duke University EC database is an Institutional Review Board-approved, prospectively enrolling database that includes sociodemographic and clinicopathologic data, comorbid conditions, adjuvant treatments, and cancer outcomes.

Subjects were eligible if they underwent surgery for early-stage endometrial cancer between January 1998 and February of 2015. Inclusion criteria were stage 1 endometrioid endometrial adenocarcinoma with total hysterectomy and bilateral salpingo-oophorectomy, no metastasis identified in lymph nodes on adequate bilateral lymph node sampling, documented LVSI status in pathology report, and at least 24 months of clinical follow-up since surgery. Within each set of nodal basins (“side”), adequate lymph node sampling was defined as at least five pelvic lymph nodes removed, at least one pelvic lymph node removed plus aortic node sampling, or successful sentinel lymph node mapping and biopsy. The criterion of 10 non-sentinel pelvic lymph nodes (five from each “side”) to establish adequate pelvic lymph node sampling was based on the average number of lymph nodes sampled in comparable studies and the definition of adequate lymph node dissection defined in GOG 210 [11, 13, 15]. Stage of disease was determined using 2009 FIGO criteria [16]. Adjuvant treatments were administered at the discretion of the primary oncologist. Given that most recurrences are diagnosed within 2 years after treatment, a minimum of 24 months of clinical follow-up was required for study inclusion unless prior recurrence was documented [6]. Recurrence was defined by biopsy or PET-CT imaging with hypermetabolic activity concerning for recurrence. Exclusion criteria were synchronous primary malignancy, history of pelvic radiation prior to the EC diagnosis, or incomplete/inadequate surgical staging.

Clinical and histopathological characteristics were obtained from chart reviews and included age, race, stage, grade, depth of invasion (inner, middle, and outer third), high-intermediate risk (HIR) criteria, LVSI status, number of lymph nodes removed, type of adjuvant treatment, location of recurrence, date of recurrence, and date of death. High-intermediate risk criteria were defined by GOG 99 criteria (age, depth of invasion, LVSI, and grade) [6].

The primary statistical endpoint in the institutional cohort was nodal recurrence, defined as recurrence in any lymph node basin. Bivariate logistic regression models assessed predictors of nodal recurrence. Covariates included age, grade, depth of invasion, and LVSI status. Bivariate models with 95% confidence intervals (CI) and Wald significance test were adjusted for the receipt of postoperative adjuvant whole pelvic radiation. A multivariate logistic regression model was utilized to assess the predictive value of age, grade, depth of invasion, and LVSI on nodal recurrence. Chi-square tests or Fisher’s exact tests were used to compare proportions. *T* tests were used to compare differences in age. A secondary statistical endpoint was PFS which was defined as the time from surgery to first recurrence or death from any cause and was censored at the date of the last follow-up. Cox univariate and multivariate proportional hazard models were used to identify prognostic factors contributing to PFS. Covariates were previously identified prognostic indicators and included grade, depth of invasion (inner, middle, and outer), and LVSI. The Kaplan-Meier method was used to estimate PFS, and survival curves were compared using the log-rank test. Prediction of OS could not be conducted because there were too few deaths.

### NCDB retrospective cohort study

The NCDB is a database sponsored by the American College of Surgeons and the American Cancer Society. It is a national database of oncology patient outcomes comprising 70% of new cancer diagnoses from across 1500 Commission on Cancer-accredited reporting institutions.

Inclusion criteria for the NCDB cohort were surgery for early-stage endometrial cancer between 1998 and 2012, stage I endometrioid endometrial adenocarcinoma, and adequate nodal evaluation. Adequate nodal evaluation in NCDB was defined as removal of greater than 10 pelvic lymph nodes or at least one pelvic lymph node plus aortic nodes examined. The NCDB does not include information on sentinel lymph node assessment. Subjects were excluded if there were no pelvic nodes removed regardless of aortic nodal dissection. The definition of stage 1 disease was based upon 2009 FIGO criteria [16]. Clinical and histopathological data included age, race, depth of invasion (inner and outer half), LVSI, nodal evaluation, grade, high-intermediate risk, type of adjuvant treatment, and death. The primary endpoint for the NCDB analyses was OS; the NCDB does not include details regarding PFS or specific sites of recurrence.

Cox multivariate proportional hazard models were used to identify prognostic factors for OS after adjusting for the use of adjuvant whole pelvic radiation, which treats nodal basins. Covariates included grade, depth of invasion (inner and outer half), age, and LVSI. OS was defined as time from surgery to death and was censored at the date of last follow-up. The Kaplan-Meier method was used to estimate OS, and curves were compared using the log-rank test. *P* values < 0.05 were considered statistically significant. Analyses were performed using SAS version 9.4 software (SAS Institute, Inc., Cary, NC); survival plots were created using Spotfire S+ v8.1 (TIBCO, Palo Alto, CA).

## Results

LVSI was present in 17.5% (48/275) of subjects from the Duke EC database and in 13.5% (3766/27,801) of those in the NCDB. The mean age of subjects was 63 and 62 years in the Duke and NCDB studies respectively; mean ages of those with LVSI were 65 and 64 years, respectively. Demographic and histopathological characteristics and type of adjuvant treatment of the Duke and NCDB patients are displayed in Table 1. Compared to subjects without LVSI, those with LVSI in both the Duke and NCDB databases were more likely to have deep myometrial invasion, grade 3 histology, to be categorized as high-intermediate risk by GOG 99 criteria and to receive adjuvant treatment. For the Duke institutional database, the median follow-up time was 4.5 years (2.9–6.1). For NCDB, median follow-up time was 3.1 years (2.0–4.2).

**Table 1:** Demographic and clinico-pathological characteristics of endometrial cancer subjects from institutional and national endometrial cancer database

	Institutional database				National database			
	LVSI absent (*N* = 227)	LVSI present (*N* = 48)	Total (*N* = 275)	*p* value	LVSI absent (*N* = 24,035) *n* (%)	LVSI present (*N* = 3766) *n* (%)	Total (*N* = 27,801) *n* (%)	*p* value
Age mean (SD) (*n* = 275)	63.2 (10.5)	64.8 (9.8)	63.5 (10.3)	0.3	61.6 (10.5)	64.1 (9.8)	61.9 (10.4)	< 0.001
Race (*n* = 274)				0.3				0.35
White	179 (79.2%)	32 (66.7%)	211 (77.0%)		21,201 (88.2)	3336 (88.5)	24,537 (88.3)	
African American	38 (16.8%)	10 (20.8%)	48 (17.5%)		1551 (6.4)	228 (6.1)	1779 (6.4)	
Asian	2 (0.9%)	1 (2.1%)	3 (1.1%)		534 (2.2)	102 (2.7)	636 (2.3)	
Native American, Alaskan Native	1 (0.4%)	1 (2.1%)	2 (0.7%)		–	–	–	
Native Hawaiian, Pacific Islander	1 (0.4%)	1 (2.1%)	2 (0.7%)		–	–	–	
Other	3 (1.4%)	1 (2.1%)	4 (1.5%)		496 (2.1)	71 (1.9)	567 (2.0)	
Unknown	2 (0.9%)	2 (4.1%)	4 (1.5%)		253 (1.1)	29 (0.8)	282 (1.0)	
Stage (*n* = 269)				0.047				< 0.001
Ia	84 (38.0%)	11 (22.9%)	95 (35.3%)		18,713 (77.9)	1651(43.8)	20,364 (73.2)	
Ib	137 (62.0%)	37 (77.1%)	174 (64.7%)		5322 (22.1)	2115 (56.2)	7437 (26.8)	
Grade (*n* = 275)				< 0.001*				< 0.001*
1	120 (52.9%)	8 (16.7%)	128 (46.5%)		10,832 (45.1)	873 (23.1)	11,705 (42.1)	
2	77 (33.9%)	22 (45.8%)	99 (36.0%)		7165 (29.8)	1282 (34.2)	8447 (30.4)	
3	30 (13.2%)	18 (37.5%)	48 (17.5%)		2552 (10.6)	1070 (28.5)	3622 (13.1)	
Unknown	–	–	–		3486 (14.5)	541 (14.2)	4027 (14.4)	
Depth of invasion (*n* = 275)				< 0.001				
Inner 1/3	146 (64.3%)	16 (33.3%)	162 (58.9%)		–	–	–	
Middle 1/3	59 (26.0%)	17 (35.4%)	76 (27.6%)		–	–	–	
Outer 1/3	22 (9.7%)	15 (31.3%)	37 (13.5%)		–	–	–	
High-intermediate risk (*n* = 275)				< 0.001				< 0.001
No	178 (78.4%)	9 (18.7%)	187 (68%)		12,631 (52.8)	657 (17.5)	13,288 (48.0)	
Yes	49 (21.6%)	39 (81.3%)	88 (32%)		11,404 (47.2)	3109 (82.5)	14,513 (52.0)	
Adjuvant therapy (*n* = 63)				0.15**				< 0.001**
External beam radiation therapy	2 (0.9%)	8 (16.7%)	10 (3.6%)		394 (1.6)	349 (9.4)	743 (2.7)	
Vaginal brachytherapy	16 (7.1%)	14 (29.2%)	30 (10.9%)		3788 (15.7)	1520 (40.3)	5308 (19.0)	
Hormonal	8 (3.9%)	5 (14.7%)	13 (5.4%)		115 (0.5)	16 (0.4)	131 (0.5)	
Chemotherapy	3 (1.5%)	6 (17.1%)	9 (3.8%)		915 (3.8)	520 (13.8)	1435 (5.2)	
Other	1 (0.4%)	0 (0%)	1 (0.4%)		1 (0.004)	2 (0.1)	3 (0.01)	
No additional treatment	–	–	–		18,822 (78.4)	1359 (36.0)	20,181 (72.6)	

Proportions are compared using a chi-square test, and age is compared using an independent *t* test

*SD* standard deviation

*For comparing grades 1 and 2 to 3

**Excludes other categories due to small sample size in this group

### Institutional database analysis

In total, 275 subjects met the inclusion criteria for institutional database analysis. There were 184 subjects excluded for lack of adequate lymphadenectomy. Of the 275 included subjects, 32% (88/275) were classified HIR and 68% (187/275) were identified as low risk.

Among included institutional subjects, 13% (36/275) experienced a recurrence, of which 30.5% (11/36) of recurrences were in cases with LVSI and 69.5% (25/36) in cases without LVSI. The most frequent sites of recurrence were the vaginal vault (14/36; 39%), pelvic lymph nodes (8/36; 22%), and the abdomen (7/36; 19%). The rate of nodal recurrence was more than 20-fold higher in cases with LVSI (9/48; 18.8%) compared to those lacking LVSI (2/227; 0.9%; *p* <  0.001). Of the vaginal recurrences, 4/14 (28.6%) had LVSI. Of the abdominal recurrences, 3/7 (42.8%) had LVSI. There were too few vaginal vault (4/48) and abdominal (3/48) recurrences among LVSI-positive subjects to evaluate association between LVSI and these sites of recurrence in univariate analysis.

In total, there were 11 nodal recurrences: 9 subjects with LVSI and 2 subjects without LVSI. The clinical-pathological characteristics of these recurrences are described in Table 2. Nine patients had both LVSI and a nodal recurrence: 5 in pelvic nodes, 1 in both pelvic and aortic nodes, 1 in only aortic nodes, and 2 in mediastinal nodes. All 3 cancers with nodal recurrence after whole pelvic radiation recurred outside of the radiation field in either para-aortic or mediastinal lymph nodes. Table 3 summarizes predictors of nodal recurrence in bivariate and multivariate analysis among all institutional EC database subjects. Grade (*p* = 0.046) and LVSI (*p* < 0.001) were significant predictors of nodal recurrence in bivariate logistic regression models after adjusting for adjuvant whole pelvic radiation. In a multivariate logistic regression analysis, LVSI was the only significant predictor of nodal recurrence (*p* = 0.002) after adjusting for age, grade, depth of invasion, and adjuvant whole pelvic radiation. Within only the low-risk subset (*n* = 187), LVSI was associated with risk of nodal recurrence after adjusting for adjuvant whole pelvic radiation (*p* = 0.04).

**Table 2:** Clinical-pathological characteristics of 11 nodal recurrences—institutional endometrial cancer database

Nodal recurrence	Age	Stage	Grade	LVSI	HIR	Nodal assessment	Adjuvant therapy	Nodal recurrence	Concurrent recurrence sites
I	57	1B	2	Present	Y	Pelvic	Vaginal brachytherapy	Pelvic	–
II	61	1A	2	Absent	N	Pelvic	Megestrol acetate	Pelvic	Lungs
III	54	1A	1	Absent	N	Pelvic	None	Pelvic	Vaginal vault
IV	72	1B	3	Present	Y	Pelvic and PA	Pelvic EBRT	Pelvic, para-aortic	–
V	65	1A	3	Present	Y	Pelvic and PA	None	Pelvic	Vaginal vault, bone
VI	52	1B	2	Present	Y	Pelvic	None	Pelvic	–
VII	64	1B	2	Present	Y	Pelvic	None	Pelvic	Vaginal vault
VIII	77	1B	3	Present	Y	Pelvic and PA	Vaginal brachytherapy	Pelvic	Abdomen
IX	67	1B	3	Present	Y	Pelvic	Pelvic EBRT	Mediastinum	–
X	70	1A	3	Present	Y	Pelvic	Pelvic EBRT	Mediastinum	Brain
XI	65	1A	3	Present	Y	Pelvic and PA	Chemotherapy	Para-aortic	Abdomen

*LVSI* lymphovascular space invasion, *HIR* high-intermediate risk, *Y* yes, *N* no, *EBRT* external beam radiation therapy, *PA* para-aortic

**Table 3:** Logistic regression bivariate and multivariate analysis to determine predictors of nodal recurrence adjusting for adjuvant radiation using institutional EC database

Variable	Bivariate analysis			Multivariate analysis		
	OR point estimate	95% CI for OR	*p* value	OR point estimate	95% CI for OR	*p* value
Age	1.01	0.95–1.08	0.78	1.01	0.92–1.07	0.85
Grade						
Grade 2 vs grade 1	3.10	0.31–31.27	0.046*	1.31	0.12–14.90	0.22
Grade 3 vs. grade 1	11.67	1.26–108.22		4.56	0.43–46.30	
Outer 1/3 invasion	1.49	0.32–6.84	0.61	1.37	0.25–7.50	0.72
LVSI	44.2	5.0–387.5	< 0.001*	33.33	3.69–323.93	0.002*
Adjuvant EBRT	–	–	–	1.45	0.15–3.19	0.63

*LVSI* lymphovascular space invasion, *OR* odds ratio, *EBRT* external beam radiation therapy; *Significantly associated with nodal recurrence after adjusting for adjuvant radiation

**Fig. 1:**
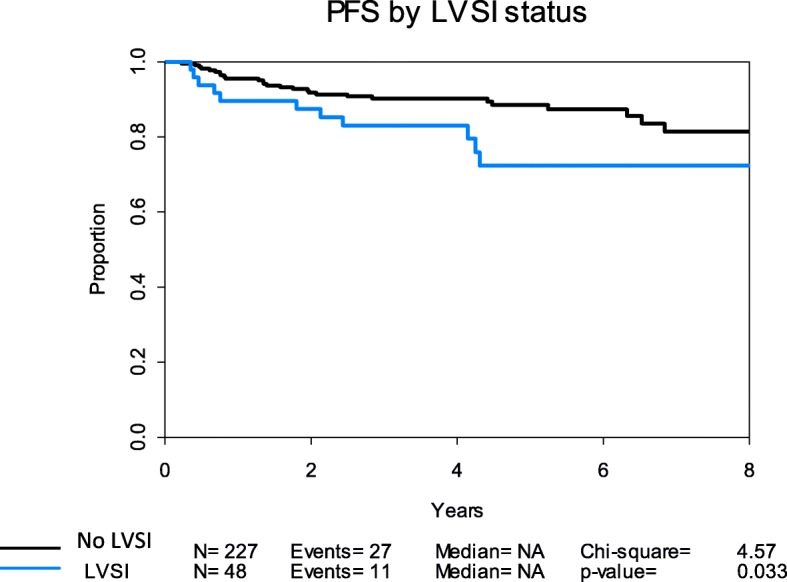
Kaplan-Meier curve for PFS (institutional EC database) in LVSI-positive compared to LVSI-negative patients

Cox univariate and multivariate analyses of covariates on PFS are described in Table 4. When compared to grades 1–2, grade 3 (HR 2.48, 95% CI 1.25–4.92) and LVSI (HR 2.12, 95% CI 1.05–4.28) were both significantly associated with shorter PFS. At 24 months, PFS was 91.8% (95% CI 88.2–95.5%) in subjects without LVSI and 87.5% (95% CI 78.5–97.4%) in subjects with LVSI. At 36 months, PFS was 90.2% (95% CI 86.3–94.3%) in subjects without LVSI compared to 83.0% (95% CI 72.9–94.5%) in subjects with LVSI. Age, depth of invasion, and high-intermediate risk criteria were not significant predictors of PFS in univariate analysis. Among only the low-risk subset (*n* = 187), LVSI was not a predictor of PFS (*p* = 1.0) in univariate analysis. In a multivariate model, only grade 3 was associated with shorter PFS (HR 2.10, CI 1.03–4.32; *p* = 0.04). A Kaplan-Meier analysis for PFS by LVSI status is displayed in Fig. 1; LVSI was associated with shorter PFS (*p* = 0.033).

**Table 4:** Cox univariate and multivariate analyses of PFS in endometrioid endometrial cancer patients using institutional EC database

Covariates	Univariate analysis			Multivariate analysis		
	Hazard ratio (HR)	95% CI for HR	*p* value	Hazard ratio	95% CI for HR	*p* value
Age	1.02	0.98–1.05	0.28	1.02	0.98–1.05	0.35
Grade						
Grade 3 vs. grades 1–2	2.48	1.25–4.92	0.01*	2.10	1.03–4.32	0.04*
Outer 1/3 invasion vs. inner 2/3	0.94	0.33–2.65	0.90	0.65	0.22–1.90	0.43
LVSI	2.12	1.05–4.28	0.04*	1.85	0.87–3.94	0.11
High-intermediate risk	1.74	0.91–3.32	0.92	–	–	–

*LVSI* lymphovascular space invasion; *Significantly associated with shorter progression free survival

The small institutional cohort size of 275 yielded too few deaths (*n* = 8/275) to analyze covariates’ effects on OS within this institutional population.

### NCDB analysis

**Fig. 2:**
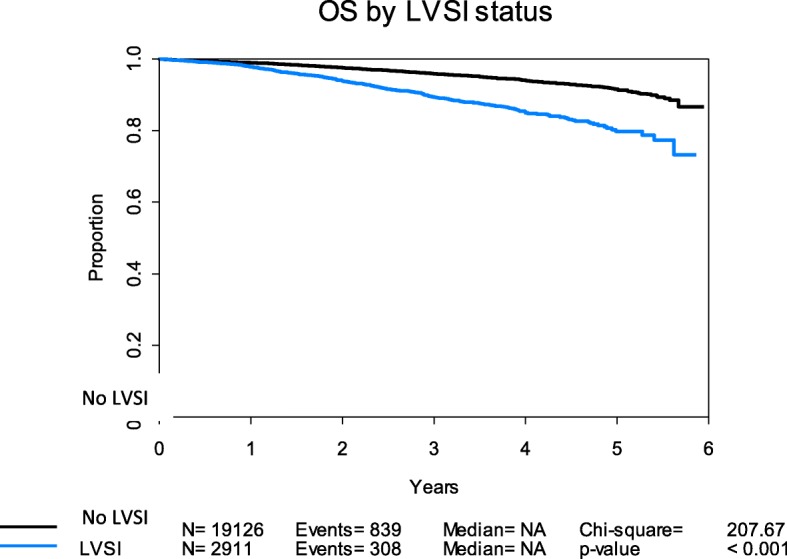
Kaplan-Meier curve for OS (National Cancer Database) in LVSI-positive compared to LVSI-negative patients

In multivariate Cox regression, grade 3 (HR 2.24, CI 1.95–2.56), outer half myometrial invasion (HR 1.33, CI 1.17–1.52), LVSI (HR 1.70, CI 1.47–1.98), and older age (HR 1.06, CI 1.05–1.07) were all significantly associated with decreased OS (all *p* < 0.001) after adjusting for the use of adjuvant whole pelvic radiation. A Kaplan-Meier curve for OS is displayed in Fig. 2; those with LVSI had shorter OS (*p* < 0.001) compared to cases lacking LVSI.

## Discussion

There have been no recent studies describing the predictive role of LVSI for nodal recurrence exclusively in stage I endometrioid EC patients with negative lymph node dissection or sentinel node biopsy. The current study identifies LVSI as a significant predictor of nodal recurrence with over 20-fold increased risk, from < 1% to over 18%. After adjusting for competing factors such as age, grade, deep myometrial invasion, and adjuvant EBRT, LVSI was an independent predictor of nodal recurrence and shorter PFS. LVSI was also a predictor of decreased OS among a much larger cohort of subjects from the NCDB when adjusting for competing covariates. These findings suggest consideration of LVSI when making decisions about adjuvant treatment of patients who have stage I disease with negative lymph node staging.

LVSI is an established risk factor for lymph node metastasis and local regional recurrence [4, 5, 7, 10]. As described by Bosse et al. utilizing PORTEC 1 and 2 data, substantial LVSI was the strongest independent prognostic factor for any pelvic recurrence, with the 5-year regional risk 15.3% with LVSI compared to 1.7% without LVSI [10]. Bosse et al. defined substantial LVSI as diffuse or multifocal LVSI present through the tumor [10]. The ideal adjuvant treatment for the small subset of patients with early-stage EC and LVSI in the presence of a negative nodal assessment, however, is the subject of much debate. This is mostly due to scant data supporting a survival benefit following adjuvant treatment [6, 10]. The PORTEC trials found that EBRT reduced the 5-year risk of pelvic recurrence in unstaged early EC patients with substantial LVSI, but did not significantly affect survival [8, 10, 14, 17]. The current evidence suggests that adjuvant therapy is beneficial in reducing risk of locoregional relapse, but the lack of survival benefit reduces clarity regarding the use of LVSI as a determinant of adjuvant therapy in early-stage endometrial cancer.

In our institutional study, nine nodal relapses occurred among cases with LVSI, two as isolated pelvic nodal recurrences following no adjuvant EBRT. These two pelvic node recurrences could potentially have been prevented by adjuvant EBRT. In order to prevent pelvic nodal recurrence in two additional patients among the 40 LVSI subjects who did not receive EBRT in our study, the number needed to treat (NNT) with EBRT to prevent one nodal recurrence is 20. This raises the question of the cost and potential side effects of external beam radiation. The PORTEC 3 was a multicenter study of 660 subjects randomized to RT versus chemoradiation [18]. Among the 329 who received radiation therapy, PORTEC 3 reports the occurrence of any adverse events at 29% (*p* < 0.0001), specifically muscle pain in 10%, hematological toxicities in 8% (*p* < 0.0001), and any gastrointestinal events in 5% [18]. Onsrud et al. found that women < 60 years old experienced a higher mortality rate after receiving EBRT, many due to an almost a doubled risk of secondary malignancy [19]. These studies highlight the tradeoffs between desired locoregional recurrence benefits and treatment toxicities when considering adjuvant treatment with EBRT.

Data suggesting that LVSI is also associated with distant recurrence highlights the need to consider systemic treatments among women with high-risk features [5, 10]. While our institutional dataset of surgical stage I cases did not have the power to examine the relationship between LVSI and distant recurrence, Bosse et al. noted that substantial LVSI was an independent prognostic indicator for distant metastasis among 259 subjects with stage IB–II EC [10]. Likewise, Gadducci et al. found that LVSI was a significant predictor of distant recurrence [5]. Emerging data in higher risk cohorts also supports systemic adjuvant therapy. For example, in the GOG 258 trial of women with advanced stage, optimally debulked EC, RT followed by chemotherapy reduced both vaginal and nodal recurrence rates compared to chemotherapy alone; however, there was no recurrence-free survival advantage [20]. Considering these data and the known toxicities of external beam radiotherapy, consideration could be given to chemotherapy treatment of those with LVSI.

Strengths of this study include the substantial incidence of LVSI in both the Duke and NCDB EC databases. The incidence of LVSI was 13.5% and 17.5% respectively in these stage I EC study populations, somewhat higher than the 10% average in patients with stage I disease and the 9.8% noted by Neal et al. among their 205 node-negative, stage I/II EC patients [3, 10]. Additionally, the Duke EC database had adequate power to establish a significant association between LVSI and nodal recurrence. Another strength is the large size of the NCDB database, which allowed us to demonstrate a significant relationship between LVSI and OS from NCDB data, a relationship that previously has been difficult to establish [8, 10, 14, 16].

Limitations of this study include the retrospective nature of the analysis. Also, the small institutional cohort size of 275 yielded too few deaths for overall survival analysis. For this reason, we supplemented our analysis using the NCDB cohort. A limitation of the NCDB database is the lack of information regarding recurrence date and location, which precluded examining associations with PFS and specific sites of recurrence such as in the lymph nodes. Finally, the Duke EC database is a prospectively enrolling study requiring informed consent for participation. As such, not all patients treated for EC at our institution were eligible for this analysis.

## Conclusion

In conclusion, our data supports that among women with stage I endometrioid EC who had a negative nodal assessment, LVSI is a significant prognostic factor for nodal recurrence, but is not an independent predictor of PFS. In a national cohort, LVSI is associated with lower OS after adjusting for competing covariates such as age, grade, depth of invasion, and adjuvant EBRT. Based on our study and previously published data, LVSI increases the risk of both nodal and distant recurrences and may be used to guide clinical decision-making when considering adjuvant treatments to reduce the risk of nodal relapse. However, the risks vs. benefits of adjuvant treatment should be carefully considered in patients with stage I EC. Given the current limited data for survival benefit in patients who receive adjuvant EBRT as well as its known risks, additional studies are needed to determine the role of adjuvant therapy in the subgroup of patients with early-stage EC with LVSI [6, 8, 10].

## Abbreviations

EBRT: External beam radiation therapy
EC: Endometrial cancer
GOG: Gynecology Oncology Group
LVSI: Lymphovascular space invasion
NCDB: National Cancer Database
OS: Overall survival
PFS: Progression-free survival
